# Spatio-temporal comprehensive measurement of China’s agricultural green development level and associated influencing factors

**DOI:** 10.1371/journal.pone.0288599

**Published:** 2023-08-04

**Authors:** Liang Cheng, Yulong Gao, Xinglong Dai

**Affiliations:** 1 College of Geography and Environmental Science, Northwest Normal University, Lanzhou, China; 2 Key Laboratory of Resource Environment and Sustainable Development of Oasis, Gansu Province, Lanzhou, China; Qufu Normal University, CHINA

## Abstract

Green development is an inevitable trend in the modernization of agriculture and rural areas, and promoting the green development of agriculture has always been an important measure for China’s sustainable growth. However, due to the influence of diverse regional environments and the wide range of landscapes in China, a largely agricultural country, China is facing ongoing challenges in improving the overall level of agricultural green development and narrowing regional differences, which has recently garnered worldwide attention. This study aims to measure and analyze the agricultural green development level of 30 provinces in China (Tibet, Hong Kong, Macao, and Taiwan are not included in the target areas of this research due to a lack of data). Here, we applied GIS technology, an entropy-TOPSIS (technique for order of preference by similarity to ideal solution) model, quantitative analysis methods such as global spatial autocorrelation analysis, coldspot and hotspot analysis, and a spatial Durbin model to construct measurement models and index systems, after which we performed a comprehensive spatiotemporal analysis of China’s agricultural green development level. Furthermore, the present study also analyzed the factors that influence agricultural green development in China. The present study demonstrated that: (i) between 2005 and 2020, China’s overall level of agricultural green development exhibited a fluctuating upward trend, with significant improvement and enhancement in most provinces. However, the overall level of China’s agricultural green development remains low, and differences at the provincial level are particularly prominent, with the main regions displaying the following descending development pattern: Eastern > Central > Western regions. (ii) The level of China’s agricultural green development shows clear signs of spatial aggregation, characterized by spatial dependence and heterogeneity. Although this phenomenon is gradually weakening over time, the high levels of agricultural green development in the eastern regions and low levels in the western regions are likely to persist in the near future. (iii) Green agricultural structure, technology supply, agricultural mechanization level, and arable land area are the key factors influencing China’s level of agricultural green development. Among these factors, technology supply, agricultural mechanization level, and arable land area have the largest direct impact, whereas green agricultural structure has a positive spatial spillover effect on the level of agricultural green development. Technology supply has both a positive direct impact and a negative indirect impact on the level of agricultural green development. Therefore, further improving technology supply and agricultural mechanization level can directly promote China’s agricultural green development.

## 1 Introduction

China is a global superpower with a population of over one billion inhabitants, and China’s agriculture is a fundamental sector of its national economy. Therefore, the stable development of agriculture is the cornerstone of China’s agricultural and rural modernization, rural revitalization, and agricultural prowess [1]. However, due to China’s rapid industrialization, a growing number of people have begun to realize that although modern agriculture brings about high labor productivity and generates a wide array of basic products and commodities, it also leads to a series of environmental impacts such as soil erosion, excessive use of chemical fertilizers and pesticides, and environmental pollution. Therefore, the green development of agriculture must be urgently promoted in China. Agricultural modernization has historically served as the foundation of national development [2], and agricultural production is unique in that it is strongly determined by both natural and economic factors. The main premise of agricultural green development is to achieve economic, social, and ecological benefits while minimizing environmental impacts. Green development relies on the use of various modern technologies and follows a scientific and evidence-driven process for sustainable development [3–5]. Promoting the green development of agriculture is not only an economic transformation process that involves the adjustment of agricultural infrastructure and production modes but also entails a revolution of consumer behavior and consumption patterns [6]. In 2015, the United Nations Sustainable Development Summit adopted the 2030 Agenda for Sustainable Development and established the goals of global sustainable development, which emphasized the importance of sustainable agriculture and considered the development of sustainable agriculture as a crucial objective. In 2022, the Food and Agriculture Organization of the United Nations released the report titled “The State of Food and Agriculture 2022,” which introduced the vital concept of green agricultural development to promote the transformation of the agricultural food system and deepen the sustainable development of agriculture [7]. Green agriculture has evidently become a crucial theme in the current era of development. Therefore, the pursuit of green agriculture is a critical component of global efforts toward sustainable development, and it has become a significant research topic that is attracting worldwide attention.

The report of the 20^th^National Congress of the Chinese Communist Party (CCP) proposes to firmly promote the revitalization of rural industries and ecology, firmly hold the red line of 1.8 billion mu (120 million hectares) of cultivated land, gradually rebuild all permanent basic farmland into high-standard farmland, implement in-depth agricultural revitalization measures, strengthen agricultural technology and equipment support, and develop facility agriculture and green agriculture [8, 9]. There are still considerable differences in the level of agricultural development in different regions of China, which seriously hinders the progress of green agricultural development. Therefore, China is constantly adjusting and optimizing its policies and strategies [10]. In 2021, the Ministry of Agriculture and Rural Affairs, the National Development and Reform Commission, and the Ministry of Science and Technology jointly issued the 14^th^Five-Year National Agricultural Green Development Plan, which clearly proposes to promote the decision-making and deployment of agricultural green development to accelerate the green transformation of agriculture and to continuously improve the environmental quality of rural regions. In 2022, General Secretary Xi Jinping also emphasized the importance of green agricultural development in his report to the 20^th^National Congress of CPP, highlighting its important position in promoting rural revitalization [11]. In 2023, the No. 1 central document emphasized “strengthening the construction of agricultural infrastructure, strengthening the support of agricultural technology and equipment, and accelerating the promotion of green agricultural development” [12]. The green development of agriculture has evidently become an important strategic plan for national growth. Therefore, the creation of new strategies to further promote the green development of agriculture and narrow the regional development gap has become a key research topic in the process of building a modern socialist country in every possible aspect.

To conduct agricultural green development research, we must first pay attention to agricultural green development measurement because countries have their own focus on understanding the concept of green development. Therefore, the choice of evaluation methods is different. For example, sustainable development, green development, production efficiency, and other aspects must be considered when assessing these methods, which complicates the selection criteria of criteria for the evaluation of agricultural green development. Therefore, this study sought to measure and analyze the agricultural green development level of 30 provinces of China (Tibet, Hong Kong, Macao, and Taiwan are not included in the target areas of this research due to a lack of data). Here, we applied GIS technology, an entropy-TOPSIS (technique for order of preference by similarity to ideal solution) model, quantitative analysis methods such as global spatial autocorrelation analysis, coldspot and hotspot analysis, and a spatial Durbin model to construct measurement models and index systems, after which we performed a comprehensive spatiotemporal analysis of China’s agricultural green development level. Furthermore, the present study also analyzed the factors that influence agricultural green development in China. The findings of this study are significant both theoretically and practically. From a theoretical perspective, this study can supplement and improve the agricultural geography research system, enrich our theoretical understanding of human economic geography and sustainable development, and contribute to our overall knowledge of regional disparities and agricultural sustainability. In terms of its practical implications, this study can provide a scientific basis to further improve the level of agricultural green development and narrow regional differences in China. The present study can also provide theoretical support and a decision-making basis for the implementation of rural revitalization, agricultural power planning, and land use planning, which are among China’s most important policy priorities.

## 2 Literature review

### 2.1 Evaluation indexes

Since the concept of green development was extended to the agricultural area, the academic community has conducted extensive research on the creation of agricultural green development indicator systems, yielding numerous research outcomes. For instance, Kanter et al. [13] developed an evaluation system for green agricultural growth that comprises five dimensions: agronomy, environment, socio-economics, agricultural product variety, and human nutrition, with a focus on the synchronization of human development and agricultural output. Quintoro-Angel et al [14] highlighted the comprehensiveness of the assessment system and developed an agricultural green development evaluation framework from three dimensions: social, economic, and ecological. Alfsen et al [15] proposed the concept of "Norwegian Natural Resource Accounting" in a study of Norwegian natural resources and created a system of green national accounting based on it. In a study of the ecological environment, Johns Brooke et al [16] introduced the notion of a "green index" based on economic development, examined the US states based on the index system, and proposed a new index system. The index’s states were graded, and the authors advocated for the government’s involvement in economic development and environmental improvement. Boix-Fayos C. et al. [17] and EC [18] proposed indicators such as land resources, arable land use, agricultural resources, and pesticide fertilizer application based on economic development to measure the green development of agriculture. While assessing international research trends, Liu Y. et al. [19], Jianbo SHEN [20], Zhang Naiming [21], and others proposed indicators such as land resources, arable land use, agricultural resources, and pesticide fertilizer application based on economic development, which are used to measure the green development of agriculture. Combined with the characteristics of China’s agricultural development, we examined the resources, ecology, and rural areas of China and constructed an evaluation index system of China’s agricultural green development from four dimensions: resource conservation, environmental friendliness, rural development, and product safety. Xin Ling [8] et al. focused on green supply and industrialization from the perspective of green development, the supply of quality and benefits, and large-scale production. Through the analysis of the four industrial levels in China, the authors concluded that the level of agricultural development in China is low. Moreover, the development among regions is extremely uneven. Jia Yunfei et al. [22] developed an index framework based on five dimensions: resource utilization, economic advantages, production environment, ecosystem, and green atmosphere, and chose provincial levels to completely quantify the level of agricultural green development.

### 2.2 Influencing factors

With the continuous development of green agriculture, the academic community has conducted an increasing amount of research on the influencing factors of agricultural green development level. For example, Winsberg MD [23] explored the concentration and specialization of American agriculture, and analyzed the influencing factors of agricultural economic growth from the perspective of industrial agglomeration. Shahbaz M et al [24] and Onoja JJ [25] investigated agricultural economy and agricultural productivity, respectively, and discovered that the magnitude of financial growth has a beneficial influence on agricultural development. Ruttan VW [26] and Hayami Y [27] made important biochemical and mechanical technical advances, respectively, and proved their influence on agricultural green development from various angles. Between 1990 and 2004, Serrao A [28] conducted an agro-ecological efficiency survey in 15 European nations and argued that technical advancement is the key influencing factor impacting agro-eco-efficiency. Guo B et al. [29] used geographic detectors to identify the determinants of agricultural green total factor production efficiency, as well as geographical differences. Jin G et al. [30] introduced a decoupling analysis model to identify the relationship between CO_2_ emissions and poverty alleviation. Chen Z et al. [31] investigated the effect of environmental legislation as a moderator of the influence of agricultural technical innovation on agricultural carbon emissions. According to Liang Jun et al. [32], technical advancement is the most important influencing factor in enhancing the efficiency of green agriculture growth. Gaimei et al. [33] investigated the significant factors affecting the green development of agriculture in Northeast China based on the 2010–2019 major grain-producing areas and discovered that economic development, international trade, and information communication were significant factors promoting the green development of agriculture. According to the findings of Fang Fuqian and Zhang Yanli [34], the advancement of rural labor and technology is a crucial factor influencing agricultural total factor productivity.

Academic research on green agricultural development has yielded several discoveries. However, there are still several gaps that must be filled. Firstly, the study on the establishment of agricultural green development indicator systems is still in the stage of discussion and investigation, has not yet developed a uniform standard, and the incorporation of lacks policy-level concerns. Second, in terms of research ideas and methods, the spatiotemporal comprehensive integrated measurement research on the level of agricultural green development and the analysis of influencing factors is still relatively lacking, with the help of GIS technology and spatial analysis models, and from the two dimensions of time and space. Furthermore, while there are many studies on the degree of agricultural green development in a nation, the total level of research in a country is generally insufficient, and this study will be able to close the knowledge gap mentioned above.

## 3 Data and methods

### 3.1 Index system and data sources

Due to the large population and significant regional differences in China, achieving sustainable agriculture development requires accounting for the national conditions, highlighting resource conservation, environmental friendliness, technological progress, and promoting rural revitalization. At the same time, government policies play an important role in guiding the development of green agriculture. Therefore, based on the existing research results [35–42] and following the principles of scientificity, systematicity, effectiveness, accessibility, and operability, the present study selected green atmosphere, economic efficiency, rural development, and government policy support indicators based on sustainable development goals(SDGs) in addition to the actual characteristics of China’s regions. The evaluation index system for agricultural sustainable development constructed in this study consists of primary, secondary, and tertiary indicators. The upper-level indicators are a comprehensive summary of the lower-level indicators, whereas the lower-level indicators are a specific decomposition and support of the upper-level indicators, as detailed in Table 1.

**Table 1:** The evaluation index system of the level of China’s agricultural green development.

First-level indicator	Second-level indicator	Third-level indicator	Unit	Indicator attributes	Indicator sources
*Green atmosphere*	Green production	Fertilizer application intensity	tons/ha	-	
		Pesticide application intensity	tons/ha	-	
		Farmland plastic film coverage	tons/ha	-	
	Ecological conservation	Quantity of solid waste utilization and disposal	ten thousand tons	+	SDGs6.4
		Total amount of chemical oxygen demand emitted by agriculture (COD)	ten thousand tons	-	SDGs3.9
		Area of water and soil erosion control	thousand hectares	+	SDGs15.3
*Economic efficiency*	Development effectiveness	Land output ratio	ten thousand yuan / thousand hectares	+	SDGs2.3
		Per capita output value of agriculture, forestry, animal husbandry, and fishery	ten thousand yuan / person	+	SDGs8.1
	Resource utilization	Area of solar water heaters in rural areas	ten thousand cubic meters	+	
		Total biogas production from rural biogas digesters	ten thousand cubic meters	+	
		Replanting index	%	+	
	Development potential	Full-time equivalent of research and development (R&D) personnel	person/year	+	SDGs12.a
		Intensity of R&D expenditure	ten thousand yuan	+	SDGs12.a
		Cultural level of rural labor force (sampled according to a fixed proportion)	person	+	
*Rural development*	Human settlements environment	Rural electricity consumption	hundred million kilowatt-hour	+	
		Total length of hardened roads	ten thousand kilometers	+	SDGs9.1
		Qualification rate of rural drinking water	%	+	SDGs3.9
		Forest coverage	%	+	SDGs15.2
	Living standards	Per capita housing area	square meter	+	
		Per capita disposable income of rural residents	ten thousand yuan / person	+	SDGs10.1
*Government policy support*	Investment in agricultural development	Total amount of agricultural subsidies	ten thousand yuan	+	
		Proportion of financial expenditure on agriculture, forestry, and water affairs	%	+	
		Total amount of rural social relief	ten thousand yuan	+	SDGs10.3
	Investment in environmental protection	Proportion of investment in agricultural disaster relief and treatment	%	+	SDGs13.1
		Proportion of investment in environmental protection funds	%	+	SDGs15.a

Based on the existing research results, combined with the characteristics of China’s agricultural green development, seven types of indicators were screened: agricultural green structure [43–45], economic level [46], direct capital supply [47–49], technology supply [50, 51], talent supply [52], mechanization level [53, 54], and cultivated land area. Afterward, we constructed an index system of influencing factors that affect China’s agricultural green development level system the index selection is as follows, as detailed in Table 2.

**Table 2:** Index system of influencing factors affecting China’s agricultural green development level.

Explanatory variables	Abbreviation	Definition
*Green agriculture structure*	STR	Proportion of green agricultural output in the total agricultural output
*Economic development level*	GDP	Gross domestic product per capita in the region
*Direct funding supply*	MS	Proportion of agricultural subsidies in the total agricultural investment
*Technology supply*	TEC	Number of agricultural technical personnel
*Talent supply*	TAL	Number of undergraduate students majoring in agriculture
*Mechanization Level*	MEC	Total power of agricultural machinery of a specific year
*Cultivated land area*	ACR	Total area of cultivated land of a specific year

This study took 2005–2020 as the research period and selected indicators from 30 provinces (municipalities and autonomous regions) in China as the research samples, and analyzed the spatial correlation and influencing factors of China’s agricultural green development level. The data sources include the annual China Statistical Yearbook, the China Rural Statistical Yearbook, the China Environmental Statistical Yearbook, the provincial statistical yearbooks and statistical bulletins, as well as literature materials. Moreover, linear interpolation was used to fill in individual missing data points in the datasets.

### 3.2 Research methods

#### 3.2.1 Entropy weight TOPSIS model

The entropy-weighted TOPSIS model is used to measure the level of green development in Chinese agriculture, and the specific calculation steps are as follows [55]:

After data standardization, the weights of the indicators were determined
  Proportion coefficient:
$$V_{ij}=\frac{y_{ij}}{\sum_{i=1}^{m}y_{ij}},\;0⩽V_{ij}⩽1$$ (1)
  Information entropy:
$$e_{j}=-k\sum_{i=1}^{m}V_{ij}\ln V_{ij},k=\frac{1}{\ln(m)},k⩾0,e_{j}⩾0$$ (2)
  Redundancy of information entropy:
dj=1‐e (3)
  Indicator weight:
$$W_{j}=d_{j}/\overset{n}{\underset{j=1}{\sum }}d_{j}$$ (4)
Construct a weighted data matrix. Let the matrix Y = (yij)m×n, then multiply each column of Y by the indicator weight Wj, and obtain a new data matrix Z = (Zij)m×n
Determine the positive and negative ideal solutions. Let Z+ represent the solution with the best indicators, i.e., the positive ideal solution, and Z- represent the solution with the worst indicators, i.e., the negative ideal solution. Then:
$$\begin{array}{l} Z^{+}=\{\underset{1⩽i⩽m}{\max}Z_{ij}|j=1,2,\cdots ,n\}=\{Z_{1}^{+},Z_{2}^{+},\cdots ,Z_{n}^{+}\}\\ Z^{-}=\{\underset{1<i⩽m}{\min}Z_{ij}|j=1,2,\cdots ,n\}=\{Z_{1}^{-},Z_{2}^{-},\cdots ,Z_{n}^{-}\} \end{array}$$ (5)
Calculate the distance. Calculate the distance from the indicator values of each province to the positive ideal solution and the negative ideal solution, respectively.
$$\begin{array}{c} d_{i}^{+}=\sqrt{\sum_{j=1}^{n}{\left(Z_{ij}-Z_{j}^{+}\right)}^{2}}(i=1,2,\cdots ,m)\\ d_{i}^{-}=\sqrt{\sum_{j=1}^{n}{\left(Z_{ij}-Z_{j}^{-}\right)}^{2}}(i=1,2,\cdots ,m) \end{array}$$ (6)
Calculate the closeness of each province to the optimal solution
$$C_{i}=\frac{d_{i}^{-}}{d_{i}^{-}+d_{i}^{+}}$$ (7)

In the formula, Ci represents the closeness of each province to the optimal solution, with a value range of [0,1]. The larger the value of Ci, the closer the province is to the optimal solution.

#### 3.2.2 Global spatial autocorrelation analysis

To analyze the spatial pattern of China’s agricultural green development level, a spatial autocorrelation model was constructed using the global Moran’s I index to identify statistically significant clustering or dispersion in the distribution of agricultural green development across all regions. The specific formula is as follows:

$$I=\frac{n}{S_{0}}\frac{\sum_{i=1}^{n}\sum_{j=1}^{n}w_{i,j}z_{i}z_{j}}{\overset{n}{\underset{i=1}{\sum }}z_{i}^{2}}$$ (8)

where zi is the deviation of the attribute value of sample i from its mean value, namely(*x_i_*−*X*), W_i,j_ is the spatial weight between samples i and j,n is the total number of samples, and S_0_ is the set of all spatial weights.


$$S_{0}=\overset{n}{\underset{i=1}{\sum }}\overset{n}{\underset{j=1}{\sum }}w_{i,j}$$ (9)

The statistical z-scores are calculated as follows:

$$z_{I}=\frac{I-E[I]}{\sqrt{V[I]}}$$ (10)

for which:

$$\begin{array}{c} E[I]=-1/(n-1)\\ V[I]=E[I^{2}]-E{\left[I\right]}^{2} \end{array}$$ (11)

#### 3.2.3 Hotspot and coldspot analysis

The Getis-Ord G* index was used to explore the local spatial autocorrelation of the level of China’s agricultural green development, as well as to investigate the clustering characteristics and patterns of high/low values. The index was calculated as follows:

$$G_{i}^{*}=\frac{\sum_{j=1}^{n}W_{ij}x_{j}}{\sum_{j=1}^{n}x_{j}}(j\mathrm{neq}i)$$ (12)

where x represents the sample value of the j-th evaluation object, n represents the number of evaluation objects, and W_i,j_ represents the spatial weight matrix. A significant positive (negative) G value indicates that the region units with high (low) observation values are clustered around region i, which belong to hot (cold) spot areas.

#### 3.2.4 Spatial Durbin model

The spatial panel model encompasses both spatial and temporal effects, making the spatial regression model more realistic [56]. Therefore, the spatial Durbin model (SDM) was used to analyze the factors that influence China’s agricultural green development level using the following formula:

$$\begin{array}{l} IGDI_{it}=\alpha +\rho W\cdot IGDI_{it}+\beta_{1}OPE+\beta_{2}IND+\beta_{3}ENE+\\ \;\beta_{4}\ln GDP+\beta_{5}\ln TAL+\beta_{6}\ln TEC+\beta_{7}REG+\beta_{8}STR+\\ \;\theta_{1}W\cdot OPE+\theta_{2}W\cdot IND+\theta_{3}W\cdot ENE+\\ \;\theta_{4}W\cdot \ln GDP+\theta_{5}W\cdot \ln TAL+\theta_{6}W\cdot \ln TEC+\\ \;\theta_{7}W\cdot REG+\theta_{8}W\cdot STR+u_{i}+\lambda_{t}+\mu_{it} \end{array}$$ (13)

where IGDI_it_ represents the index of green development in Chinese agriculture for province i and period t; α is the intercept; ρ represents the spatial regression coefficient; β represents the regression coefficient of the explanatory variable; θ represents the regression coefficient of the spatial lag of the explanatory variable; W is the spatial weight matrix. W is equal to 1 if there is a common border between regions, otherwise, W = 0. Due to the unique geographical location of Hainan and to ensure the scientific rigor of this study, its common border with Guangdong was adjusted. Here, u_i_ represents the spatial fixed effects; λ_t_ represents time fixed effects. If u_i_ and λ_t_ are related to the explanatory variables, a fixed effects model was selected. Otherwise, a random effects model was selected. Moreover, μit represents the spatial autocorrelation error term.

## 4 Comprehensive spatiotemporal measurement of agricultural green development level

### 4.1 Analysis of agricultural green development at the provincial level

From 2005 to 2020, the level of China’s agricultural green development exhibited a fluctuating upward trend (Table 3). However, based on the national average level, the overall growth rate only increased from 0.255 in 2005 to 0.288 in 2020, an increase of merely 0.023. The average value of the national agricultural green development level index is 0.277, meaning that China’s agricultural green development level has ample room for improvement. Upon analyzing the agricultural green development level index of each province, some provinces initially exhibited an increasing trend followed by a decreasing trend, whereas most provinces maintained a continuous growth trend, suggesting that the level of agricultural green development in most provinces has been steadily increasing. Among all provinces, Shandong exhibited the highest level of agricultural green development, with an average value of 0.576. Shandong has a mild climate, abundant sunlight, continuous heat, and both rain and heat during the same season. It is suitable for the growth and development of various crops and is one of the birthplaces of the agriculture industry in China. This province has insisted on scientific and innovation-driven technological leadership and has prioritized the stimulation of endogenous power in rural areas, the promotion of rural development, and the continuous improvement of agricultural quality, efficiency, and competitiveness. During the "13^th^ Five-Year Plan" period, Shandong became the first province in China with an agricultural total output value exceeding one trillion yuan. Jiangsu and Guangdong provinces ranked second and third respectively, with their average scores being 0.446 and 0.405. Jiangsu is located in the Yangtze River Delta region, and this province is characterized by a flat terrain and many rivers and lakes. The proportion of plains and water surface in this province ranks first in China. Its agricultural production conditions are unique in the country, making it the main producing area of agricultural and grain products, as well as being an advantageous area for producing high-quality and low-gluten wheat in China. Moreover, Jiangsu has abundant scientific and technological vitality and advanced ecological and environmental protection concepts. This province vigorously promotes clean agricultural production, being the first in the country to launch a policy on clean energy drying and to carry out experimental projects on the construction of ecological farmland. Guangdong is China’s largest foreign trade province and a major province for the import and export of agricultural products. It has long supported the alliance of the production, education, and research sectors composed of foreign-related enterprises and agricultural research institutions. By focusing on a variety of professional fields, it has improved the quality and level of agricultural production and kept the level of green development of its agriculture at the forefront of the country. Zhejiang and Sichuan provinces have average values greater than 3.5. Zhejiang is among the highest-yielding grain-producing areas in China, with its rice yield per unit area being at the highest level in China. Agricultural technology is advancing rapidly, and the level of agricultural mechanization is relatively high. Sichuan is a large agricultural province located on the Chengdu Plain with fertile soil and good production conditions. Additionally, Sichuan’s agricultural production mode has quickly transformed from traditional agriculture to modern agriculture, and rural infrastructure construction has been improved. Therefore, its agricultural green development level is constantly improving. The agricultural green development level of the remaining provinces was relatively low, with an average level between 0.181 and 0.328.

**Table 3:** China’s agricultural green development level indexes.

Province	2005	2006	2007	2008	2009	2010	2011	2012	2013	2014	2015	2016	2017	2018	2019	2020	AVG	Rank
*Beijing*	0.313	0.330	0.305	0.330	0.329	0.310	0.321	0.313	0.319	0.317	0.300	0.323	0.308	0.306	0.293	0.283	0.312	9
*Tianjin*	0.184	0.190	0.184	0.182	0.184	0.187	0.193	0.215	0.225	0.224	0.218	0.233	0.188	0.175	0.234	0.152	0.198	25
*Hebei*	0.289	0.316	0.288	0.313	0.295	0.286	0.286	0.330	0.292	0.283	0.283	0.339	0.293	0.280	0.268	0.327	0.298	12
*Shanxi*	0.152	0.203	0.292	0.200	0.241	0.182	0.189	0.186	0.179	0.174	0.175	0.197	0.172	0.176	0.168	0.177	0.191	28
*Neimenggu*	0.214	0.337	0.321	0.248	0.246	0.288	0.303	0.273	0.288	0.291	0.303	0.297	0.272	0.299	0.310	0.268	0.285	14
*Liaoning*	0.346	0.302	0.314	0.285	0.307	0.286	0.296	0.316	0.314	0.299	0.281	0.267	0.268	0.300	0.267	0.265	0.295	13
*Jilin*	0.195	0.227	0.208	0.217	0.211	0.197	0.196	0.191	0.230	0.187	0.187	0.190	0.179	0.185	0.184	0.243	0.202	24
*Helongjiang*	0.178	0.220	0.231	0.214	0.196	0.197	0.228	0.222	0.242	0.244	0.238	0.219	0.222	0.272	0.228	0.234	0.224	21
*Shanghai*	0.324	0.329	0.322	0.352	0.370	0.336	0.335	0.321	0.319	0.308	0.305	0.327	0.308	0.328	0.298	0.286	0.323	8
*Jiangsu*	0.399	0.426	0.417	0.440	0.476	0.429	0.441	0.437	0.451	0.453	0.456	0.475	0.452	0.460	0.459	0.458	0.446	2
*Zhejiang*	0.350	0.325	0.324	0.374	0.389	0.348	0.408	0.345	0.358	0.358	0.376	0.401	0.382	0.394	0.388	0.359	0.367	4
*Anhui*	0.261	0.196	0.245	0.207	0.229	0.259	0.224	0.234	0.237	0.269	0.244	0.257	0.261	0.246	0.256	0.268	0.243	17
*Fujian*	0.261	0.267	0.289	0.343	0.265	0.266	0.306	0.303	0.309	0.323	0.320	0.321	0.304	0.308	0.320	0.332	0.302	11
*Jiangxi*	0.194	0.208	0.216	0.257	0.217	0.229	0.282	0.225	0.271	0.224	0.227	0.257	0.249	0.244	0.258	0.296	0.241	18
*Shandong*	0.512	0.531	0.520	0.592	0.608	0.577	0.614	0.615	0.598	0.600	0.595	0.579	0.567	0.583	0.558	0.571	0.576	1
*Henan*	0.272	0.323	0.298	0.322	0.326	0.299	0.309	0.308	0.316	0.325	0.329	0.346	0.338	0.369	0.364	0.377	0.326	7
*Hubei*	0.249	0.277	0.291	0.302	0.313	0.293	0.298	0.293	0.322	0.357	0.315	0.334	0.332	0.338	0.324	0.341	0.311	10
*Hunan*	0.276	0.299	0.341	0.324	0.320	0.288	0.323	0.296	0.372	0.319	0.330	0.348	0.367	0.346	0.358	0.339	0.328	6
*Guangdong*	0.375	0.361	0.347	0.369	0.385	0.402	0.372	0.403	0.400	0.400	0.427	0.452	0.422	0.461	0.447	0.449	0.405	3
*Guangxi*	0.282	0.277	0.295	0.277	0.257	0.242	0.243	0.228	0.246	0.247	0.243	0.282	0.264	0.238	0.242	0.241	0.256	16
*Hainan*	0.212	0.209	0.219	0.225	0.221	0.212	0.281	0.211	0.231	0.235	0.225	0.231	0.243	0.222	0.252	0.220	0.228	20
*Chongqing*	0.155	0.205	0.237	0.199	0.173	0.177	0.206	0.188	0.211	0.194	0.194	0.207	0.193	0.202	0.208	0.201	0.197	26
*Sichuan*	0.344	0.325	0.319	0.351	0.356	0.344	0.347	0.362	0.347	0.357	0.367	0.383	0.412	0.407	0.398	0.402	0.364	5
*Guizhou*	0.208	0.209	0.200	0.194	0.175	0.164	0.170	0.166	0.172	0.181	0.188	0.206	0.195	0.191	0.234	0.224	0.192	17
*Yunnan*	0.255	0.260	0.256	0.333	0.267	0.257	0.256	0.279	0.279	0.281	0.285	0.324	0.302	0.294	0.286	0.330	0.284	15
*Shaanxi*	0.238	0.233	0.220	0.241	0.237	0.229	0.241	0.220	0.236	0.230	0.230	0.239	0.234	0.223	0.231	0.233	0.232	19
*Gansu*	0.169	0.162	0.228	0.221	0.160	0.164	0.171	0.190	0.160	0.158	0.165	0.175	0.193	0.188	0.185	0.203	0.181	29
*Qinghai*	0.215	0.200	0.208	0.221	0.226	0.201	0.211	0.222	0.215	0.218	0.230	0.215	0.220	0.228	0.237	0.249	0.220	22
*Ningxia*	0.071	0.067	0.098	0.081	0.075	0.164	0.075	0.073	0.081	0.078	0.082	0.087	0.088	0.095	0.091	0.100	0.088	30
*Xinjiang*	0.150	0.179	0.278	0.232	0.178	0.200	0.192	0.214	0.207	0.216	0.206	0.239	0.215	0.216	0.219	0.210	0.209	23
*AVG*	0.255	0.266	0.277	0.282	0.274	0.267	0.277	0.273	0.281	0.278	0.277	0.292	0.281	0.286	0.286	0.288	0.277	

**Fig 1:**
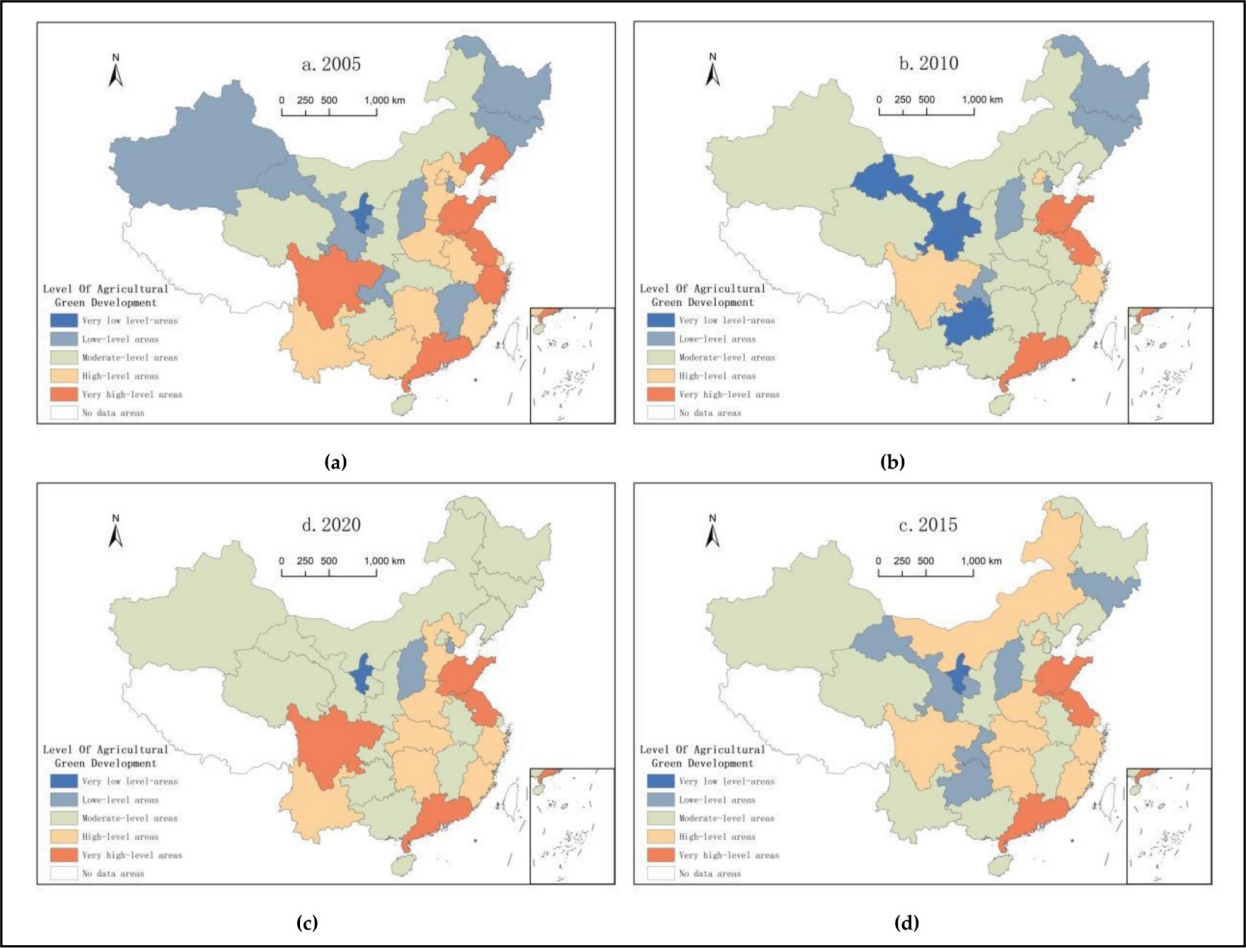
Spatial distribution of China’s agricultural green development level.

The present study utilized the GIS10.4 software to more accurately reflect the spatial distribution characteristics of China’s agricultural green development level at the provincial level, after which we selected China’s agricultural green development level indexes in the years2005, 2010, 2015, and 2020as nodes for data visualization (Fig 1). The 30 provinces in China were divided into five categories based on their level of agricultural green development: very low-level areas, low-level areas, moderate-level areas, high-level areas, and very high-level areas.

From 2005 to 2020, China’s agricultural green development level showed significant changes in its spatial distribution as follows: (1) the number of very high-level areas increased to four, which were mainly distributed in the Sichuan Basin, as well as eastern and southern coastal areas; (2) the number of high-level areas did not change much, with only one additional area. The spatial distribution changed from point-like dispersion to a linear distribution in Hebei, Henan, Hubei, Hunan, Sichuan, Zhejiang, Fujian, and other provinces; (3) the number of moderate-level areas increased to 16, which were mainly distributed in Xinjiang, Gansu, Qinghai, Inner Mongolia, Chongqing, Shaanxi, Guangxi, Anhui, Jiangxi, and other provinces in a sheet form, showing a spatial evolution trend extending from the western and northeastern regions to the south; (4) the low-level areas showing a shrinking trend were first discretely distributed across the Xinjiang, Gansu, Heilongjiang, Jilin, Chongqing, Jiangxi, and Shanxi provinces, and were then dotted in Ningxia, Shanxi, and Tianjin provinces; (5) very low-level provinces appeared intermittently, and finally changed from Ningxia towards low-level areas.

Overall, the changes in China’s provincial agricultural green development level were quite apparent. The provinces with the highest levels of development were mainly concentrated in the southwest and eastern coastal areas, whereas the provinces with low levels of development are mainly distributed in the central and western regions, as well as in some southern regions.

### 4.2 Analysis of the level of agricultural green development in the three major regions

**Fig 2:**
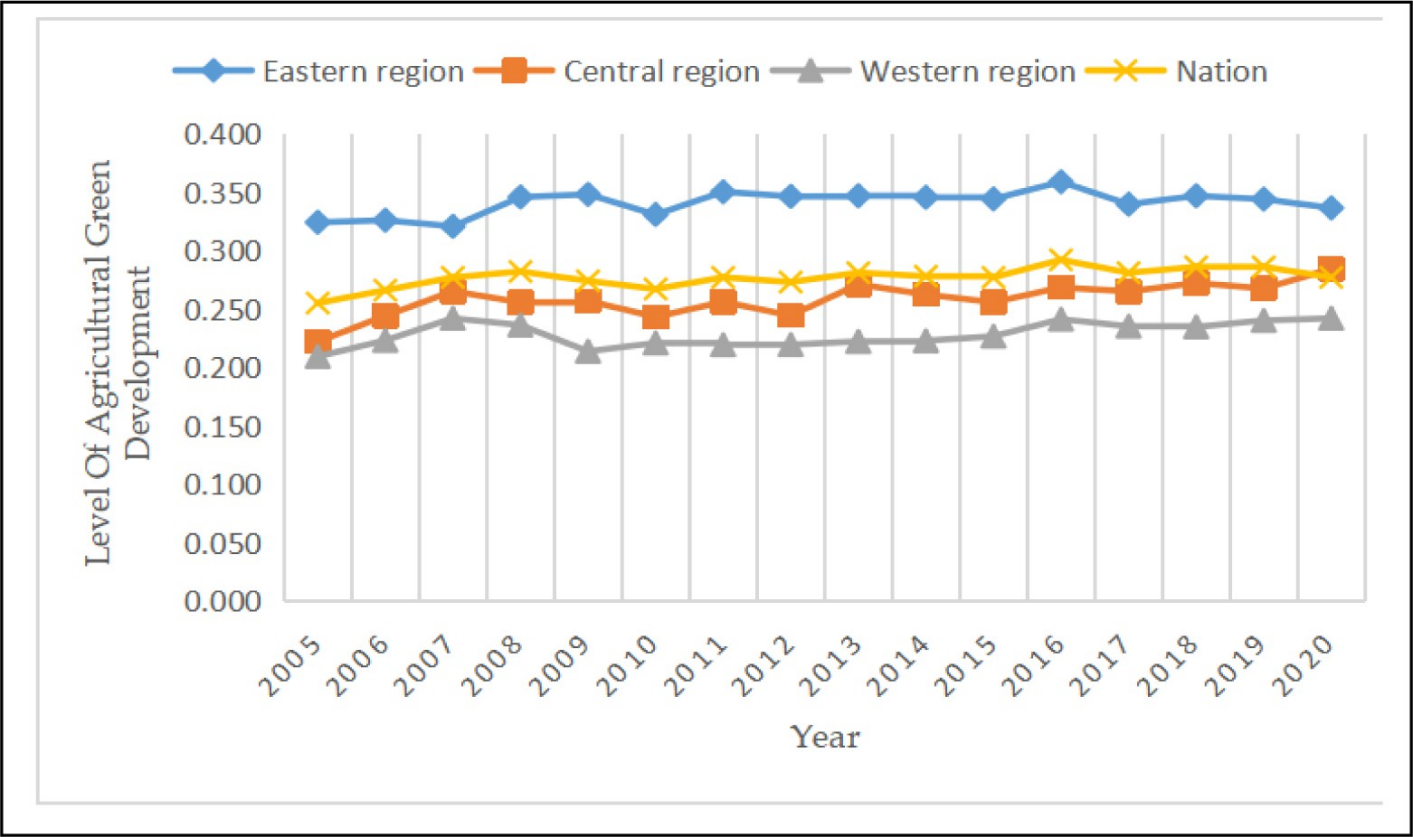
Changes in the agricultural green development level trends of the three major regions.

The eastern, central, and western regions are the three major economic regions in China. From 2005 to 2020, the agricultural green development level indexes of these regions showed a steady upward trend (Fig 2). The eastern region exhibited the highest level of agricultural green development, followed by the central region, and finally the western region. Therefore, the agricultural green development level of China exhibited a ladder-like trend from east to west. The agricultural green development level in the eastern region was significantly higher than the national average, whereas the central and western regions were lower than the national average. Specifically, the agricultural green development level index in the eastern region rose from 0.324 in 2005 to 0.359 in 2015 and then dropped to 0.336 in 2020, with an average value of 0.341, which was higher than the national average of 0.277. The agricultural green development level index in the central region rose from 0.222 in 2005 to 0.284 in 2020, showing a fluctuating upward trend, with an average value of 0.258, which was lower than the national average. The agricultural green development level index in the western region rose from 0.209 in 2005 to 0.242 in 2007, then dropped to 0.214 in 2009, and then continued to rise to 0.242 in 2020, with an average value of 0.228, which was lower than the national average.

The agricultural green development growth trend in the eastern and central regions was more apparent, whereas that in the western region was slower, suggesting that the level of agricultural green development was closely related to the economic development level and scientific and technological innovation capabilities of the region. The index first exhibited an upward trend followed by a decrease, indicating that China has made remarkable progress in terms of agricultural green development. However, the central and western regions still face many challenges such as the implementation of adequate ecological environment governance, which largely restricts the green development of agriculture in these regions.

### 4.3 Local high and low-value cluster analysis

**Fig 3:**
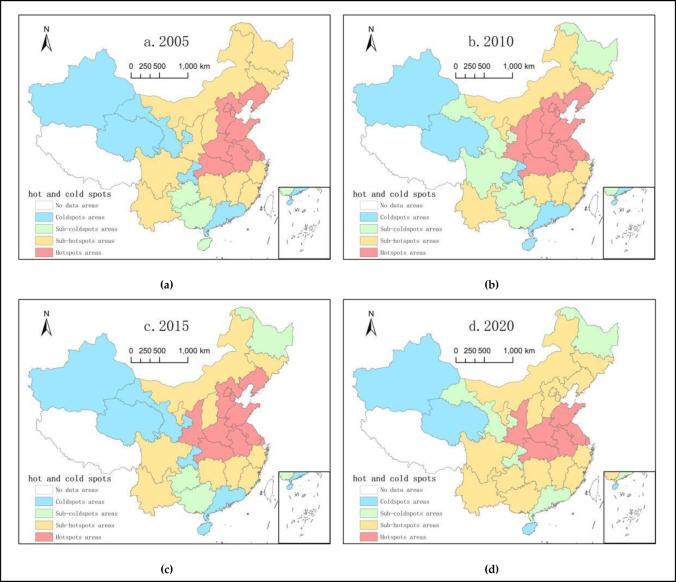
Spatial evolution of coldspots and hotspots of China’s agricultural green development index.

The present study utilized the GIS10.4 software and selected China’s agricultural green development level indexes in the years2005, 2010, 2015, and 2020as nodes to conduct a local high and low-value cluster analysis of China’s agricultural green development level. The analysis relies on the locations of coldspots, sub-coldspots, hotspots, and sub-hotspots to reflect the local distribution patterns of agricultural green development levels (Fig 3).

From 2005 to 2010, there were significant changes in the overall spatial distribution of coldspots and hotspots in regard to China’s agricultural green development. The number of hotspots increased from 9 to 11, with Shaanxi and Shanxi turning from sub-hotspots to hotspots, whereas the rest of the provinces remained unchanged. At this point, the hotspots were still concentrated in the central and eastern coastal areas, whereas the number of sub-hotspots decreased from 14 to 8, which were distributed in a belt-like pattern around the hotspots. The area of sub-coldspots expanded from the central region towards the southern region, and Gansu, Sichuan, and Heilongjiang changed from sub-hotspots to sub-coldspots. The number of coldspots remained unchanged and were mainly distributed in Xinjiang, Qinghai, Chongqing, Guangdong, Hainan, and other regions. From 2010 to 2015, there were little changes in the overall spatial distribution of coldspots and hotspots in China’s agricultural green development level, with the number of hotspots remaining mostly unchanged except for Shanxi, in which the number of hotspots decreased. The number of sub-hotspots increased from 8 to 10, with Sichuan shifting from a sub-coldspot to a sub-hotspot, and Shanxi shifting from a hotspot to a sub-hotspot. Moreover, there were distributed in patches in the northern and southwestern regions. The number of sub-coldspots decreased to three, with Heilongjiang, Guizhou, and Guangxi remaining unchanged. Gansu shifted from a sub-coldspot to a coldspot, and Sichuan shifted from a sub-coldspot to a sub-hotspot, with a more scattered spatial distribution. The number of coldspots did not change significantly, with only one addition in Gansu, and the spatial distribution was mainly concentrated in Xinjiang, Qinghai, Gansu, Chongqing, Guangdong, Hainan, and other regions. From 2015 to 2020, the overall spatial distribution of coldspots and hotspots in China’s agricultural green development level changed significantly. The number of hotspots decreased from 11 to 7, with Liaoning, Hebei, Beijing, and Tianjin shifting from hotspots to sub-hotspots. Moreover, these hotspots were concentrated in the central and eastern coastal areas. The number of sub-hotspots increased to 16, with the addition of Guizhou and Guangxi to the previously identified regions, and these regions were concentrated in the northern and southern parts of China. The area of sub-coldspots decreased in Guizhou and Guangxi, while it increased in Gansu, Chongqing, and Guangdong, and the spatial distribution became more discrete. The number of coldspots decreased to three, which were mainly distributed in Xinjiang, Qinghai, and Hainan.

### 4.4 Global spatial autocorrelation analysis

Overall, the level of agricultural green development in China’s provinces exhibited significant spatial agglomeration and distribution characteristics with obvious spatial dependence. Over time, hotspots have shown a tendency to expand and concentrate, indicating that the degree of agglomeration in the spatial distribution of China’s agricultural green development is increasing, particularly in the central and eastern coastal areas. The sub-coldspot areas were mainly concentrated in the inland and underdeveloped regions of the northwest, and the level of agricultural green development exhibited a stepwise decrease from east to west, displaying regular spatial distribution characteristics with high levels in the east and low levels in the west.

The present study utilized the ArcGIS10.4 software to compute the global Moran’s l index of China’s agricultural green development level index from 2005 to 2020 (Table 4). The index values were consistently positive across the study period, with z-test values exceeding the critical value of 1.65. Our results demonstrated a significant correlation at the 0.1 level and passed the significance test with a confidence level of 90%, suggesting that China’s agricultural green development level displays a positive spatial autocorrelation. At the provincial scale, there was an overall aggregation pattern, where provinces with high levels of agricultural green development tended to be adjacent to other provinces with similarly high levels, and vice versa. Examining the global Moran’s I index’s trend over time revealed a fluctuating pattern from 2005 to 2020, indicating that the degree of spatial autocorrelation is unstable. This finding suggests that there is a certain degree of spatial agglomeration and distribution of provinces with high or low levels of agricultural green development, which fluctuate over time.

**Table 4:** Global Moran’s I index of China’s agricultural green development level.

Year	Moran’sI	P	Q
*2005*	0.163769	2.418081	0.015603
*2006*	0.190392	2.759732	0.005785
*2007*	0.191367	2.787451	0.005312
*2008*	0.157704	2.362941	0.018131
*2009*	0.187534	2.736304	0.006213
*2010*	0.173259	2.568874	0.010203
*2011*	0.163137	2.438425	0.014751
*2012*	0.161793	2.427045	0.015222
*2013*	0.163825	2.440262	0.014677
*2014*	0.164895	2.452921	0.014170
*2015*	0.128927	1.999297	0.045576
*2016*	0.144577	2.183193	0.029022
*2017*	0.125056	1.939282	0.052467
*2018*	0.102241	1.661046	0.096788
*2019*	0.115801	1.824379	0.068095
*2020*	0.103806	1.674477	0.094037

## 5 Analysis of influencing factors of agricultural green development

The study on the spatial distribution and correlation of agricultural green development levels demonstrates the existence of spatial interactions among the agricultural green development of various provinces in China, and our findings highlighted the occurrence of significant spatial agglomeration and spatial heterogeneity. Considering the current state of China’s agricultural green development, an analysis of the external environment faced by green development was conducted. Furthermore, a spatial Durbin model was used to investigate the influence of multiple factors on the level of agricultural green development and its spatial spillover effects. These findings can provide valuable insights that would enable the implementation of precise and rational adjustments to agricultural green development, thus aiding in its sustainable progress.

There are two types of effect selection methods for panel data analysis. After conducting the Hausman and LM tests, this study selected the fixed effect model. According to the estimated results, the Wald test and LR test could not simplify the spatial Durbin model. The R2 value was 0.952, and the spatial overflow item was 0.185, which passed the 1% significance test, suggesting the occurrence of spatial spillover effects in agricultural green development. Therefore, the fixed-effect spatial Durbin model was chosen. Using the level of agricultural green development as the response variable and the influencing factors as the explanatory variables, the spatial Durbin model was then applied, with relevant results shown in Table 5.

**Table 5:** Estimation results of the spatial Durbin model.

Variable	Coefficent	Z	P	Variable	Coefficent	Z	P
*STR*	0.0006	1.80	0.071	W*STR	0.0016	2.48	0.013
*lnGDP*	0.0016	0.26	0.794	W*lnGDP	-0.0009	-0.10	0.921
*MS*	-0.0007	-1.02	0.310	W*MS	-0.0019	-1.49	0.137
*lnTEC*	0.0131	2.11	0.035	W*lnTEC	-0.0229	-2.56	0.010
*lnTAL*	0.0007	0.12	0.905	W*lnTAL	-0.0046	-0.60	0.546
*lnMEC*	0.0117	2.18	0.029	W*lnMEC	0.0032	0.27	0.788
*lnACR*	0.0130	2.51	0.012	W*lnACR	-0.0030	-0.26	0.797

(i) The coeffi(i) The coefficient rho of the lagged dependent variable reflects the impact of geographic factors on the green development of agriculture. Its value is 0.185 and passes the 1% significance test, indicating that there are spatial spillover effects and spatial dependence in the green development of agriculture in China. Geographic factors have a significant positive effect on the green development of agriculture in China, which means that changes in the green development of agriculture in neighboring areas will cause corresponding changes in this area. In fact, neighboring areas have similar agricultural development levels, which is conducive to mutual learning and the establishment of a reference for the green development of agriculture in each area, ultimately leading to the convergence of green agricultural development in adjacent regions. (ii) The agricultural structure (STR) has both a positive direct impact coefficient and a positive spatial lag coefficient, passing the significance tests at 10% and 5%, respectively. This can be explained by the fact that the development of green agriculture in neighboring provinces may result in a preference for their green agricultural products over local traditional agricultural products, resulting in an increase in the level of green agricultural development in neighboring provinces. (iii) The technology supply (TEC) has a positive direct impact coefficient and a negative spatial lag coefficient, both of which passed the 5% significance test. This can be attributed to the fact that scientific and technological innovation can accelerate the development of green agriculture and promote the level of green agricultural development. The technology supply is a specific case of positive externalities, which is not a benefit obtained within the economic activity itself, nor is it a benefit obtained by the users of the products of that activity. This benefit is external to the economic activity itself and creates external socioeconomic benefits. The increase in technological supply in neighboring provinces may weaken the research and development capabilities of this province, but it will play an important role in the green development of agriculture nationwide.(iv) Mechanization level (MEC) has a positive direct impact coefficient, which passed the 5% significance test, and the spatial lag coefficient was positive but not significant. This is because the development of agricultural mechanization can not only improve the living standards of farmers and increase the labor rate of agricultural production, but also narrow the gap between urban and rural areas, improve the overall level of agriculture and rural economy, and significantly promote the development of agriculture, rural areas, and farmers. In turn, this can comprehensively improve the level of green agricultural development. Agricultural machinery is generally large, with a narrow range of spatial movement. However, some small machinery could flow into neighboring provinces to promote the green development of agriculture in neighboring provinces, thus significantly impacting agricultural development. (v) Cultivated land area (ACR) has a positive direct impact coefficient, which passes the 5% significance test, and the spatial lag coefficient is negative but not significant. The reason is that cultivated land is the foundation of agricultural development, and a certain amount of high-quality cultivated land is a prerequisite for promoting agricultural development. When the amount of cultivated land in neighboring provinces is too high, its agricultural products will occupy the market of the province to a certain extent, making it difficult for people to realize the importance of developing green agriculture. However, the government’s macro-control strategies and the market are relatively balanced, resulting in insignificant spillover effects.

In the effect decomposition of the spatial Durbin model, the existence of spatial spillover effects means that the coefficients of each factor cannot be independently explained as the impact on the green development of agriculture. Therefore, partial differential decomposition was performed to better explain the direct and indirect effects of each influencing factor (Table 6). In terms of direct effects, the elastic coefficients of STR, TEC, MEC, and ACR were positive and passed the significance test. Among them, technology supply, mechanization level, and cultivated land area have the largest direct effects, with values of 0.012, 0.011, and 0.013, respectively. Technology supply passed the significance test at a 10% level, whereas mechanization level and cultivated land area passed the significance test at a 5% level. These are the most important factors affecting the level of green development of China’s agriculture. Given the unlikelihood that there will be a large-scale increase in the cultivated land area in the near future, the main purpose should be to maintain the existing cultivated land. Therefore, every 1% increase in the level of technology supply and mechanization will increase the level of agricultural green development by 0.012% and 0.011%, respectively. In terms of indirect effects, the elastic coefficient of STR is positive and passed the significance test at a 5% level.

**Table 6:** Estimation of direct and indirect effects of the spatial Durbin model.

Variable	Gross effect	Z	Direct effect	Z	Indirect effect	Z
*STR*	0.0026***	3.49	0.0007	1.97	0.0020**	0.00
*lnGDP*	0.0008	0.09	0.0015	0.26	-0.0007	-0.02
*MS*	-0.0031**	-1.78	-0.0008	-1.12	-0.0024	-0.01
*lnTEC*	-0.0122	-1.31	0.0121*	1.99	-0.0243	-0.04
*lnTAL*	-0.0047	-0.76	0.0004	0.06	-0.0051	-0.02
*lnMEC*	0.0186	1.23	0.0120**	2.25	0.0066**	-0.02
*lnACR*	0.0122*	0.77	0.0130	2.49	-0.0009	-0.03

Note:***P <

Note:***P < 0.01

**P < 0.05

* P < 0.1.

The green agricultural structure has a significant positive spatial spillover effect on the level of agricultural green development. This is mainly due to the high transportation distance and cost of agricultural products, as people pay more attention to health and prefer green agricultural products. In turn, the development of green agriculture in neighboring provinces promotes the local improvement of green agricultural levels. The elasticity coefficient of TEC was negative and passed the significance test at a 5% level, indicating that technology supply has both a positive direct effect and a negative indirect effect. The accumulation of scientific talent in neighboring provinces may lead to the loss of scientists in other less developed provinces, which is not conducive to improving the level of green agricultural development. However, since the technology supply is a specific case of positive externalities, although the increase in technological supply may not benefit the development of green agriculture in neighboring provinces, it will improve China’s overall research and development level. Overall, the increase in technological supply will significantly enhance China’s level of green agriculture development. In terms of the total effect, the elastic coefficient of the STR is positive, passing the significance test at a 1% level, indicating that the green agricultural structure plays a significant role in promoting the green development of China’s agriculture. The elasticity coefficient of direct capital supply (MS) was significantly negative and passed the significance test at a 5% level, indicating that direct agricultural capital subsidies have a significant inhibitory effect on the green development of agriculture in the study area. Based on these findings, we propose that free technical support, professional guidance, or indirect assistance should be provided instead of fund subsidies to promote the green development of agriculture in China.

## 6 Conclusions and discussion

### 6.1 Conclusions

From 2005 to 2020, the overall level of China’s agricultural green development showed a fluctuating upward trend. The level of agricultural green development in most provinces had been significantly improved but the overall level was still low. The differences on the provincial scale were more obvious. The higher-level areas of agricultural green development were mainly concentrated in the eastern and southern coastal developed areas and Sichuan, whereas the lower-level areas were mainly distributed in the western and northern inland underdeveloped areas. The level of agricultural green development in the three major regions rose, but the increase rate was small, and the overall spatial development pattern of the eastern, central, and western regions decreased sequentially.

China’s agricultural green development level exhibited clear spatial agglomeration and distribution characteristics, showing obvious spatial dependence and heterogeneity. Provinces with high levels of agricultural green development tended to be adjacent to other provinces with similarly high levels, whereas provinces with low levels of agricultural green development tended to be adjacent to other provinces with similarly low levels. Over the study period, this phenomenon tended to weaken over time, but the overall pattern of high levels of agricultural green development in the eastern regions and low levels in the western regions is likely to persist in the near future.

The level of China’s agricultural green development is primarily influenced by the green agricultural structure, technology supply, agricultural mechanization level, and arable land area. The direct effects of technology supply, mechanization level, and arable land area are the most significant, and the green agricultural structure exhibits a positive spatial spillover effect on the level of agricultural green development. Although technology supply has both positive direct and negative indirect effects on the level of agricultural green development, the increase and improvement of technology supply and mechanization level can directly promote the development of China’s agricultural green development level.

### 6.2 Discussion

According to our findings, China’s level of green agriculture development still has great room for improvement, with uneven development between regions and a need for further improvement in the coordination level of green agriculture development. Based on this premise, some scholars have proposed their views. For example, Li Zhou [57] proposed that providing ecological compensation funds by area would not incentivize the participants. Instead, the level of green agriculture development can only be effectively improved by creating and enhancing the self-generating ability, symbiotic ability, and harmonious ability of green agriculture development. Wang Xuemei and Ma Dexue [58] reported that implementing the concept of green development and ecological modernization is crucial to green agriculture development, whereas Xin Ling and An Xiaoning [59] proposed that green development should lead the high-quality development of agriculture in economically developed regions of eastern China, thus promoting the high-quality development level of agricultural products in western regions. Our findings highlight the importance of adhering to the principle of localized and classified strategies, as well as the promotion of regional green agriculture development policies according to the local conditions of agriculture resources, economic development foundation, and ecological environment type. These measures would allow decision-makers to make the best use of comparative advantages and form economically developed regions with distinctive features. At the same time, it is important to prioritize the development of the eastern regions and actively promote the development of low-level green agriculture development provinces in western regions, provide policy guidance in terms of talent, finance, and technology to promote green agriculture development in the central and western regions, narrow the regional differences, achieve coordinated development of green agriculture among regions, and improve China’s level of green agriculture development. Based on the research results, when formulating and implementing policies related to agricultural green development, attention should be given to the following factors:

- The western region needs to have a reasonable plan and structure for the green agricultural industry. This can be achieved by supporting the creation of high-quality agricultural product brands and cultivating green brands. It is also important to increase safety supervision of green food products, raise market awareness of geographical indication products, promote green concepts and food, and encourage the integration and development of green agriculture, food processing industries, the production service industry, and the Internet.
- The central region needs to continuously boost agricultural modernization. This can be achieved by increasing investment in the intelligent agricultural machinery industry, strengthening the construction of high-end intelligent agricultural machinery, promoting the deep integration of agricultural machinery and agronomy, vigorously promoting agricultural mechanization and intelligence, and providing scientific and technological support for agricultural modernization.
- The eastern region has sufficient innovative vitality and should actively build an agricultural science and technology innovation system. This can be achieved by building an agricultural science and technology innovation network incorporating both centralization and division through optimization and integration. Reshaping the agricultural science and technology innovation system of the entire industry chain by strengthening node links can promote agricultural technology through the development of social services. Agricultural scientific research and development can be improved by increasing investment, reforming mechanisms, and improving education quality. Appropriate policy support for agricultural scientific research institutions should be provided to accelerate the development of technology innovation alliances for green agricultural development and increase scientific and technological assistance to the central and western regions.
- Cultivated land is the foundation of China´s agricultural development, and the entire country needs to implement arable land protection measures that stabilize quantity, improve quality, and protect ecology. This can be done by simultaneously implementing high-standard farmland construction and high-efficiency water-saving irrigation overall planning. Theoretical innovation of basic farmland protection should be strengthened, basic farmland protection laws and regulations should be established and improved, permanent basic farmland should be scientifically delineated, effective protection of land space should be implemented, and intensive use of land should be realized. Moreover, land development, reclamation, and consolidation should be increased, and a cultivated land reserve system should be established. The balance between the quantity and quality of cultivated land occupation and compensation should be maintained and the ecological conditions of cultivated land should be protected.

Despite the important insights gained from our findings, our study had some limitations that need to be addressed in future research:

- Our study only focused on the narrow definition of agriculture (planting industry) and did not include forestry, animal husbandry, and fishery in the scope of green agricultural development. Therefore, further exploration and improvement are necessary to have a more comprehensive view of green agriculture.
- The selection of key indicators needs to be improved. Although this study included some indicators such as the rate of harmless treatment of rural waste, the growth rate of green food enterprise certification, and the comprehensive utilization rate of straw, reaching data-driven conclusions is difficult due to the lack of available data. Therefore, future studies should focus on collecting more data and using more robust indicators.
- The interpolation method was used to fill in the gaps in the data, which may have affected the measurement results. Therefore, it is important to collect more accurate and complete data for future research.
- Some indicators such as network application and the level of industrialization could not be included due to a lack of quantitative methods. Therefore, future research should explore more quantitative methods to improve the selection of independent variable indicators of influencing factors. Overall, addressing these limitations will contribute to strengthening the reliability and validity of future research on green agricultural development.
- Finally, Green agricultural development is a social and complex development concept combining economic, social, technological, policy and other factors. The academic community has not formed an authoritative and fixed index system, because any index system is a process of continuous exploration in a specific social environment. This study also draws on previous relevant studies and combines the social characteristics of China’s agricultural development. Focusing on the special national conditions of China and exploring and innovating the index system, it is inevitable that there are unreasonable points in the process, which will be further improved by our research group in the future.

## Supporting information

S1 File (ZIP)
